# Biopsy Morphometrics as Predictors of Treatment Response in Primary Nephrotic Syndrome

**DOI:** 10.1016/j.xkme.2025.101181

**Published:** 2025-11-07

**Authors:** Bartholomeus T. van den Berge, Jitske Jansen, Quinty Leusink, Sanne Kleuskens, Sharon Bootsman, Anne-Els van de Logt, Coralien Vink, Jack FM. Wetzels, Bart Smeets, Rutger J. Maas

**Affiliations:** 1 Department of Nephrology, Radboud Institute for Molecular Life Sciences, Radboudumc, Nijmegen, The Netherlands; 2 Department of Pathology, Radboud Institute for Molecular Life Sciences, Radboudumc, Nijmegen, The Netherlands; 3 Department of Pediatric Nephrology, Radboud Institute for Molecular Life Science, Radboudumc, Nijmegen, The Netherlands; 4 Institute for experimental medicine and systems biology, Uniklinik RWTH Aachen, Aachen, Germany

**Keywords:** Primary nephrotic syndrome, podocytes, glomerulosclerosis, proteinuria remission, predictors

## Abstract

**Rationale & Objective:**

Clinical outcome of primary nephrotic syndrome (PNS) is highly variable, and predicting an individual patient’s treatment response remains difficult. PNS is characterized by means of podocyte injury and loss. We hypothesized that histologic parameters related to podocyte depletion predict treatment response.

**Study Design:**

Retrospective cohort study.

**Setting & Participants:**

We analyzed biopsy tissue of 106 patients with PNS (minimal change disease, N = 26; focal segmental glomerulosclerosis, N = 21; and membranous nephropathy [MN], N = 59) and 9 controls. Minimal change disease and focal segmental glomerulosclerosis were considered manifestations of the same entity, defined as idiopathic nephrotic syndrome (iNS), and analyzed as one group. Patients’ baseline clinical and follow-up data were recorded. Kidney biopsies, stained for podocyte-specific and fibrosis markers, were quantitatively analyzed.

**Predictors:**

Glomerular density, glomerulosclerosis, podocyte number, podocyte density, and cortical fibrosis.

**Outcomes:**

Complete remission (CR) and delayed treatment response.

**Analytical Approach:**

Odds ratios and receiver operating characteristic—the area under the curve (ROC-AUC) values identified predictors.

**Results:**

In patients with iNS, the respective partial remission and CR rates were 29% and 60% during a median follow-up of 40 months. The majority of patients received high-dose corticosteroid treatment. Quantitation of cortical fibrosis had the highest discriminative power (ROC-AUC value, 0.79; 95% CI, 0.655-0.923) to predict CR. Other significant predictors included podocyte density, nonsclerotic glomerular density, and percentage of nonsclerotic glomeruli.

In patients with MN, respective partial remission and CR rates were 41% and 54% during a median follow-up of 50 months. The percentage of nonsclerotic glomeruli and nonsclerotic glomerular density were predictors for CR (patients receiving immunosuppressive treatment [ROC-AUC value, 0.71; 95% CI, 0.535-0.893]; patients receiving nonimmunosuppressive treatment alone [ROC-AUC value, 0.80; 95% CI, 0.584-1.000]).

**Limitations:**

Relatively small cohorts prevented the use of covariates.

**Conclusions:**

In patients with iNS, higher podocyte density and nonsclerotic glomerular density, and lower glomerulosclerosis and cortical fibrosis predicted CR. In patients with MN, lower glomerulosclerosis and higher nonsclerotic glomerular density predicted CR. Biopsy parameters may thus be useful for estimating proteinuria outcome.

## Plain-Language Summary

Primary nephrotic syndrome (PNS) is characterized by podocyte injury and loss. According to the podocyte depletion hypothesis, the outcome of glomerular injury depends on whether the remaining podocyte pool is depleted or not. Complete remission (CR) of proteinuria is the aim of treatment in PNS and is associated with long-term preserved kidney function, but it is difficult to predict individually. We hypothesized that morphometric analysis of kidney biopsies could be used to predict proteinuria outcomes. We identified several glomerular and podocyte indices as predictors of CR on an individual level. Morphometric analysis of kidney biopsies has prognostic value and may guide treatment decision-making in the future.

## Introduction

Primary nephrotic syndrome (PNS) refers to nephrotic syndrome in the absence of underlying systemic disease. Minimal change disease (MCD), primary focal segmental glomerulosclerosis (FSGS), and membranous nephropathy (MN) are the major causes of PNS.^1^ PNS is caused by disruption of the glomerular filtration barrier, particularly because of podocyte injury.^2^^,^^3^ Podocytes are terminally differentiated epithelial cells that have no or limited capacity to divide.^4^^,^^5^ Evidence suggests that PNS may be caused by autoantibodies targeting podocyte proteins, especially PLA2R (MN) and nephrin (MCD/FSGS).^6^^,^^7^ Furthermore, podocyte depletion developed into glomerulosclerosis in experimental models.^8^ In addition, podocyte damage caused further injury to other nonaffected podocytes, resulting in a self-sustaining cycle of podocyte loss.^9^ The podocyte depletion hypothesis states that outcome of any glomerular injury depends on whether or not the podocyte pool becomes significantly depleted.^10^ Compensatory maladaptive responses, such as glomerular hypertrophy, contribute to further podocyte loss. Podocyte depletion and glomerular hypertrophy may also be involved in PNS disease progression, as exemplified by the observation of an increased glomerular tuft area as an early indicator of progression to FSGS in patients initially diagnosed with MCD.^11^ Morphometric analysis of podocyte depletion and its sequelae in kidney biopsies may be useful to predict outcomes in patients with PNS. Reduction of nephron number and glomerular density has been associated with prolonged response to corticosteroid treatment in adult MCD.^12^^,^^13^ Interestingly, estimations of podocyte density in a single biopsy slide correlated well with design-based stereologic methods.^14^ PNS treatment aims to achieve proteinuria remission, which is associated with preserved kidney function. However, it remains difficult to predict proteinuria response in an individual patient.^15^, ^16^, ^17^, ^18^, ^19^ For MN, a prognostic model has been validated previously.^20^ We hypothesized that morphometric analysis, which includes counting podocytes in kidney biopsies from patients with PNS, can be used to predict the outcome of proteinuria. To investigate this, we performed a retrospective study using archived kidney biopsy material from patients with PNS with established outcomes.

## Methods

### Clinical Characteristics and Outcome Criteria

A retrospective study was performed in adult patients with PNS who were referred to Radboudumc (a university hospital) between 2011 and 2023 for patients with MN and 2016 and 2023 for patients with iNS. Patients with PNS were identified from our clinical registry and the pathology database. Inclusion criteria included nephrotic syndrome (proteinuria ≥ 3.5 g/10 mmol and hypoalbuminemia) and histopathologic diagnosis of MCD, FSGS, or MN. Permission for the use of archived material and electronic patient data was obtained by the local ethical commission for human medical research of the Radboud University Medical Center, Nijmegen, The Netherlands (approval number: 2018-4086), waiving the need for informed consent. The study data were anonymized and collected according to the Code of Conduct for Medical Research.

Kidney biopsy tissue from patients with PNS and controls without evidence of kidney disease was used. Control kidney tissue was obtained from the unaffected part of (tumor-) nephrectomies, and periodic acid-Schiff stainings confirmed normal morphology in those sections. Controls did not have proteinuria and had no history of kidney disease before (tumor) nephrectomy. Definitions of clinical outcomes were followed as described by the Kidney Disease: Improving Global Outcomes (KDIGO) 2021 guidelines.^21^ The estimated glomerular filtration rate (eGFR) at the time of biopsy was calculated using the CKD-EPI (Chronic Kidney Disease Epidemiology Collaboration) equation.^22^ In our hospital, timed urinary measurements of low-molecular-weight proteins β-2 microglobulin and α-1 microglobulin, high-molecular-weight protein IgG, and PLA2R serum titer are used to estimate the probability of spontaneous improvement versus progression, guiding therapeutic decision-making in patients with MN.^20^ For extended methods pertaining to definitions of clinical outcomes, see Item S1: Supplementary Materials and Methods.

### Immunofluorescent Staining

Immunofluorescent stainings were performed on paraffin-embedded kidney biopsy material of patients with PNS and controls. Biopsy tissue was stained for 4',6-diamidino-2-phenylindole (DAPI) (nuclei), collagen type IV (fibrous tissue and basement membranes), Wilms’ tumor 1 (podocyte-specific cytoplasm), and Dachshund family transcription factor 1 (podocyte nuclei). For extended methods, see Item S1 of Supplementary Materials and Methods. All relevant information on macros used for immunofluorescent image analyses and the respective buffer and antibody dilutions used is listed in Tables S1 and S2, respectively.

### Podocyte, Glomerular and Cortical Morphometric Analyses

For extended methods and all relevant formulas, see Item S1. Supplementary Materials and Methods.

#### Podocyte density and number, and glomerular cell density and number

Podocyte density and glomerular cell density were calculated based on the method described by Venkatareddy et al.^14^

#### Glomerular (tuft) volume

Glomerular (tuft) volume was calculated according to the equation of Weibel and Gomez.^23^ To adjust for variations in glomerular size, we used the distribution coefficient previously reported by Denic et al.^24^

#### Glomerular density

Glomerular density was calculated according to the method used by Tsuboi et al.^25^

#### Chromotrope Aniline Blue matrix deposition as a marker for cortical fibrosis

Cortical fibrosis was calculated similarly to the method used by Jansen et al.^26^

### Statistical Analysis

MCD and FSGS were considered manifestations of the same entity, defined as idiopathic nephrotic syndrome (iNS), and analyzed as one group.^6^^,^^27^ All data are expressed as median with IQR, unless stated otherwise. Receiver operating characteristic—the area under the curve (ROC-AUC) values, logistic regression analysis, all statistical analyses, and all graphs were performed using IBM SPSS Statistics version 29 (IBM). Unpaired *t* tests or, when appropriate, 1-way analysis of variance analysis followed by Dunnett’s post hoc test were performed unless stated otherwise. Missing data were left out of individual analyses.

## Results

### Baseline Clinical and Experimental Characteristics of Study Participants

**Figure 1:**
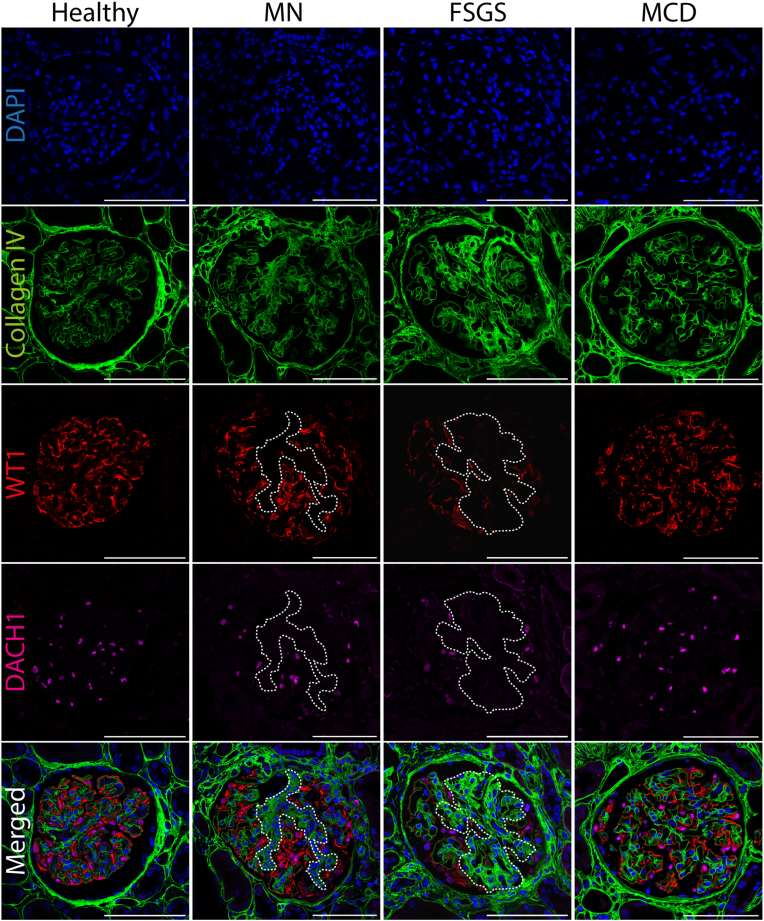
Overview of immunofluorescent staining of biopsies. Representative biopsy images of a control and of patients with membranous nephropathy (MN), idiopathic focal segmental glomerulosclerosis (FSGS), and minimal change disease (MCD), respectively. Biopsy tissue was stained for DAPI (nuclei, blue), collagen type IV (COLIV, fibrous tissue and basement membranes, green), Wilms’ tumor 1 (WT1, podocyte-specific cytoplasm, red), and Dachshund family transcription factor 1 (DACH1, podocyte nuclei, magenta). During morphometric analysis, DAPI and DACH1 were used to annotate all nuclei and podocyte nuclei, respectively. COLIV was used to annotate the glomerulus (Bowman’s capsule). WT1 was used to annotate the glomerular tuft. Dashed white lines indicate sclerotic lesions and/or mesangial hypercellularity, void of podocyte-specific cytoplasm (WT1) and DACH1^+^ podocyte nuclei. Scale bars represent 100 μm.

Among the patients who met the inclusion criteria, 59 were diagnosed with MN, 26 with MCD, and 21 with FSGS. Clinical characteristics at the time of biopsy and experimental parameters for both controls and patients with PNS are summarized in Table 1. Kidney biopsy material was obtained at the time of relapse in 19 patients (18%). No significant differences were found between clinical characteristics of patients undergoing biopsy at relapse and at the initial presentation. A representative overview of glomerular and podocyte immunofluorescent stainings for controls and PNS subtypes can be found in Fig 1. Kidney biopsies were stained for cell nuclei (DAPI), podocyte nuclei (DACH1), podocyte cytoplasm (WT1), and collagen type IV (COLIV, fibrous tissue and basement membranes). Experimental parameters in the control group for glomerular (tuft) volume (5.8 × 10^6^ and 4.3 × 10^6^ μm^3^, respectively), glomerular density (2.9 N/mm^2^), podocyte density (156 N/10^6^ μm^3^), and podocyte number per glomerular tuft (679) were in line with previously published data^14^^,^^28^^,^^29^ (Table 1). Notably, we were unable to perform immunofluorescent stainings in 26 out of 59 (44%) of the MN cohort, because commonly used Bouin’s fixative (≤ 2,016) directly interfered with the immunofluorescent stainings (Table 1).

**Table 1:** Baseline Clinical and Experimental Characteristics for Controls and Patients With PNS

Characteristic	Controls			Idiopathic Nephrotic Syndrome				Membranous Nephropathy			
	N	Median	IQR	N	Median	IQR	*P* Value vs Control	N	Median	IQR	*P* Value vs Control
Age (y)	9	68	56-72	47	57	39-72	**0.032**	59	58	45-66	0.057
Sex (% Male)	9	56%		47	57%		0.916	59	80%		0.112
Ethnicity (% White)	9	100%		47	100%		-	59	100%		-
Serum creatinine concentration (μmol/L)	9	70	62-76	42	133	76-237	**0.001**	54	92	81-108	**0.021**
eGFR (mL/min/1.73 m^2^)	9	94	81-105	43	46	24-83	**0.001**	57	77	63-92	**0.038**
Serum albumin concentration (g/L)				41	19	15-26	-	56	22	18-28	-
UPCR at biopsy (g/mmol)				40^a^	6.62	4.04-9.95	**-**	56^a^	5.54	3.80-8.00	**-**
Indication for biopsy (diagnosis/relapse)				47	37/10		-	59	50/9		-
PLA2R positive (%) PLA2R serum titer (U/mL)								59 29	76.3% 91	27.5-141.5	- -
Sclerotic glomeruli (% of total)	9	2.0%	0.0-4.2	45	5.3%	0.0-12.9	**0.001**	59	18.2%	6.5-33.3	**0.001**
Nonsclerotic glomeruli (% of total)	9	98.0%	95.9-100.0	45	94.7%	87.1-100.0	**0.001**	59	81.8%	66.7-93.6	**0.001**
Glomerular volume (10^6^ μm^3^)	9	5.8	5.0-8.2	38	5.7	4.4-6.0	0.173	55	6.4	4.7-7.5	0.920
Glomerular tuft volume (10^6^ μm^3^)^b^	9	4.3	4.1-5.1	38	4.6	3.7-6.0	0.446	32	5.6	4.2-7.2	**0.008**
Glomerular cell number (N)^b^	9	2,925	2,612-3,470	38	2,710	1,967-3,294	0.397	31	2,309	1,800-2,813	0.056
Nonpodocyte glomerular cell number (N)^b^	9	2,238	1,739-2,786	38	2,094	1,692-2,725	0.954	31	2,082	1,591-2,470	0.542
Podocyte number per glomerular tuft (N)^b^	9	679	585-844	38	367	233-524	**0.001**	31	240	169-367	**0.001**
Glomerular cell density (N/10^6^ μm^3^)^b^	9	450	410-478	38	451	403-531	0.779	31	333	314-375	0.219
Nonpodocyte glomerular cell density (N/10^6^ μm^3^)^b^	9	305	261-315	38	369	290-441	**0.046**	31	285	266-323	0.685
Podocyte density (N/10^6^ μm^3^)^b^	9	156	123-186	38	83	56-125	**0.001**	31	48	35-60	**0.001**
Glomerular density (N/mm^2^)	9	3.0	2.6-3.2	44	2.5	2.1-3.0	0.345	58	2.8	2.2-3.7	0.218
Nonsclerotic glomerular density (N/mm^2^)	9	2.9	2.5-3.2	44	2.3	1.5-2.6	0.064	58	2.3	1.4-2.9	0.194
Cortical fibrosis (μm^2^/μm^2^)	8	0.103	0.077-0.157	46	0.171	0.127-0.214	**0.022**	52	0.170	0.125-0.228	**0.001**

Continuous data are expressed as median (IQR). For patients’ biopsies, all glomeruli were analyzed for biopsies with a glomerulus count of N ≥ 4. Student’s *t*-test or χ^2^-test was performed for statistical comparison. Statistically significant *P* values (≤0.05) are stated in bold.

Abbreviations: eGFR, estimated glomerular filtration rate; IQR, interquartile range; PNS, primary nephrotic syndrome; UPCR, urinary protein creatinine ratio.

a All patients had nephrotic range proteinuria at the time of presentation (≥3.5 g/10 mmol). Proteinuria was not always measured at the time of biopsy.

b In older biopsies, the use of Bouin’s fixative prevented successful immunofluorescent stainings, and subsequent parameters could not be computed. Contemporary biopsies fixed with formalin could be analyzed.

PNS was histologically characterized by means of a higher percentage of sclerotic glomeruli, lower podocyte number/density, and increased cortical fibrosis compared with controls (Table 1). Patients with FSGS had statistically significantly higher levels of (global + focal segmental) glomerulosclerosis (20% vs 5%) and cortical fibrosis (20% vs 15%) compared with patients with MCD (*P* = 0.005 and *P* = 0.04, respectively), but were similar in all other parameters. Patients with MCD were similar to controls regarding glomerulosclerosis and cortical fibrosis (*P* = 0.10 and *P* = 0.09, respectively). Interestingly, mean glomerular tuft volume was significantly increased in patients with MN (but not in iNS) when compared with controls (Table 1). Taken together with similar levels of glomerular cell numbers/densities, this may indicate the presence of compensatory hyperfiltration.^30^

### Treatment Response Predictors in Patients With PNS

#### iNS

**Figure 2:**
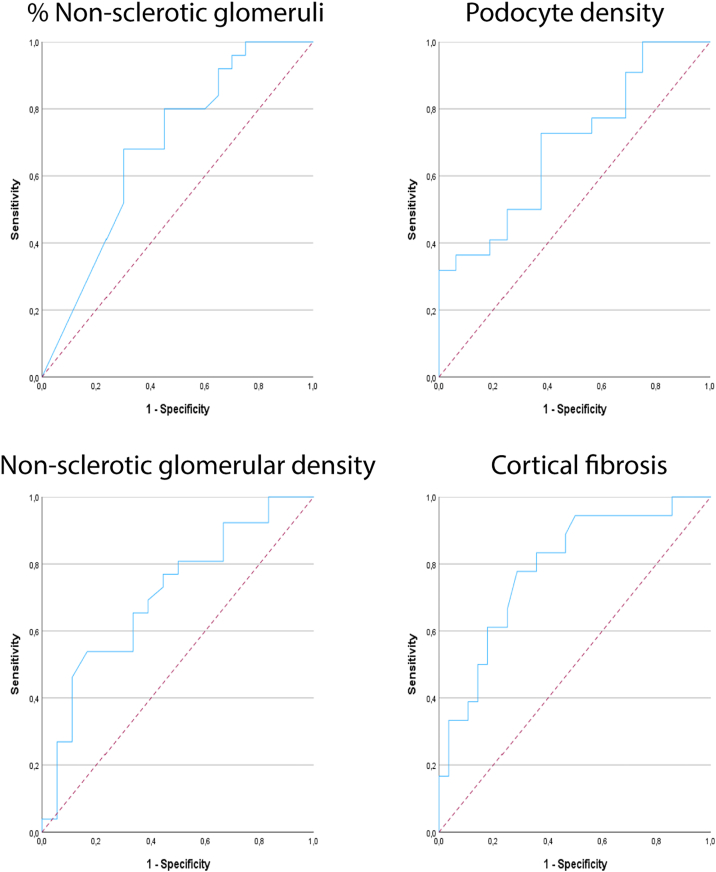
Receiver operating characteristic (ROC) curves for prognostic accuracy of reaching complete remission (CR) in patients with idiopathic nephrotic syndrome (iNS). Areas under the curve were as follows: percentage of nonsclerotic glomeruli, 0.69 (95% CI, 0.524-0.846; *P* = 0.03); podocyte density, 0.69 (95% CI, 0.525-0.861; *P* = 0.04); nonsclerotic glomerular density, 0.71 (95% CI, 0.551-0.866; *P* = 0.02); cortical fibrosis, 0.79 (95% CI, 0.655-0.923; *P* = 0.001). Dashed lines indicate the trendline. Abbreviations: CI, confidence interval

Clinical outcome measures and treatment regimens are summarized in Table 2. In the iNS cohort, with a median follow-up time of 39.8 months, patients had a median time to remission of 1.5 months. During follow-up, the overall remission rate was 89% (60% PR and 29% CR). The majority (87.2%) of patients received immunosuppressive (IS) treatment, mostly with initial prednisone monotherapy (55.3%). No statistically significant differences were observed in time to remission (*P* = 0.24), nor did treatment outcome (PR/CR%) differ significantly between treatment regimens (*P* = 0.78 and *P* = 0.69 for PR and CR, respectively). Follow-up duration was similar between patients who reached CR versus those who did not reach CR (*P* = 0.19). Using logistic regression analysis, the percentage of (non-) sclerotic glomeruli, podocyte density, nonsclerotic glomerular density, and the amount of cortical fibrosis were significantly associated with the outcome of CR (Table 3). For the percentage of nonsclerotic glomeruli, the OR was 1.063 (95% CI, 1.006-1.124; *P* = 0.03). At a threshold for the percentage of nonsclerotic glomeruli of ≥ 95%, positive predictive value (PPV) and negative predictive value (NPV) were 73% and 57%, respectively. For podocyte density, the OR was 1.021 (95% CI, 1.001-1.040; *P* = 0.04). At a threshold for podocyte density of N ≥ 80/10^6^ μm^3^, PPV and NPV values were 68% and 50%, respectively. Similarly, for nonsclerotic glomerular density, the OR was 2.403 (95% CI, 1.072-5.386), *P* = 0.03). At a threshold for nonsclerotic glomerular density of ≥ 2.2 N/mm^2^, PPV and NPV values were 72% and 58%, respectively. For cortical fibrosis, the OR was 0.841 (95% CI, 0.743-0.952), *P* = 0.006). At a threshold for cortical fibrosis of ≤19.5%, PPV and NPV values were 74% and 73%, respectively. We plotted an ROC curve for the prognostic accuracy of the percentage of nonsclerotic glomeruli, podocyte density, nonsclerotic glomerular density, and the amount of cortical fibrosis. ROC-AUC values were 0.69 (95% confidence interval [CI], 0.524-0.846; *P* = 0.03), 0.69 (95% CI, 0.525-0.861, *P* = 0.04), 0.71 (95% CI, 0.551-0.866; *P* = 0.02) and 0.79 (95% CI, 0.655-0.923, *P* = 0.001) for the percentage of nonsclerotic glomeruli, podocyte density, nonsclerotic glomerular density, and the amount of cortical fibrosis, respectively (Fig 2).

**Table 2:** Clinical Outcome Measures in Patients With Primary Nephrotic Syndrome

Characteristic	iNS			Clinical Outcome	Membranous Nephropathy			Clinical Outcome
	N	Median	IQR	Median Time to Remission (IQR) + (% Reaching PR/CR)	N	Median	IQR	Median Time to Remission (IQR) + (% Reaching PR/CR)
Time to remission (mo) (% reaching PR/CR/NR)	47	1.5 (29%/60%/11%)	0.8-5.4		59	9.8 (41%/54%/5%)	3.5-15.6	
Time to follow-up (mo)	47	39.8	12.7-61.9		59	50.1	37.5-63.0	
Treatment response KDIGO (fast/slow)	47	29/18						
Time interval between biopsy and immunosuppressive treatment (mo)	41	1.0 ± 0.6			35	4.5	2.0-10.5	
Immunosuppression received (%)	41	87.2%			35	59.3%		
No immunosuppressive treatment (%)	6	12.8%		1.3 (0.6-3.1) + (100/50%)	24	40.7%		5.0 (1.5-13.3) + (92%/58%)
Monotherapy (%)	31	66.0%			7	11.9%		
Prednisone (%)	26	55.3%		1.5 (0.8-6.0) + (85/54%)	2	3.4%		17.8 (-) + (100/100%)
Tacrolimus (%)	3	6.4%		0.8 (-) + (100/67%)	2	3.4%		13.0 (-) + (100/33%)
Rituximab (%)	2	4.3%		2.0 (-) + (100/50%)	3	5.1%		13.0 (-) + (100/33%)
Combination therapy (%)	10	21.3%			28	47.5%		
Prednisone + Tacrolimus (%)	4	8.5%		1.0 (0.3-1.0) + (100/75%)				
Prednisone + MMF (%)	6	12.8%		4.9 (1.1-13.3) + (83/67%)				
Prednisone + cyclophosphamide (%)					19	32.2%		10.0 (4.0-17.5) + (95/53%)
Prednisone + cyclophosphamide + RTX (%)					6	10.2%		8.5 (3.8-33.1) + (83/50%)
Tacrolimus + RTX (%)					2	3.4%		7.0 (-) + (100/50%)
Prednisone + cyclophosphamide + Tacrolimus + RTX (%)					1	1.7%		- (-) + (0/0%)

Percentages (%) are given as a percentage of total; the time interval between biopsy and treatment is given as mean ± SD for the iNS cohort; Slow responders were defined as having a treatment response ≥ 16 weeks.

Abbreviations: CR, complete remission; iNS, idiopathic nephrotic syndrome; IQR, interquartile range; MMF, mycophenolate mofetil; NR, nonremission; PR, partial remission; RTX, rituximab.

**Table 3:** Logistic Regression Analysis for Predicting CR and Slow Treatment Response in Patients With iNS

Characteristics	CR 95% CI				KDIGO Slow Response 95% CI			
	Odds Ratio	*P* Value	Lower	Upper	Odds Ratio	*P* Value	Lower	Upper
Age (y)	0.987	0.441	0.956	1.020	0.974	0.125	0.942	1.007
Sex (% Male)	1.391	0.583	0.429	4.515	1.277	0.689	0.386	4.228
Ethnicity (% White)	-	-	-	-	-	-	-	-
Serum creatinine concentration (μmol/L)	0.997	0.311	0.992	1.002	1.001	0.626	0.996	1.006
eGFR (mL/min/1.73 m^2^)	1.006	0.513	0.989	1.022	0.999	0.873	0.982	1.015
Serum albumin concentration (g/L)	0.975	0.553	0.896	1.060	0.933	0.155	0.849	1.026
UPCR (g/mmol)	0.999	0.995	0.850	1.175	1.050	0.560	0.891	1.237
Sclerotic glomeruli (% of total)	0.940	**0.031**	0.889	0.994	1.016	0.349	0.983	1.051
Nonsclerotic glomeruli (% of total)	1.063	**0.031**	1.006	1.124	0.984	0.349	0.952	1.018
Glomerular volume (10^6^ μm^3^)	0.882	0.519	0.602	1.292	0.794	0.262	0.531	1.188
Glomerular tuft volume (10^6^ μm^3^)	1.014	0.949	0.652	1.578	0.765	0.255	0.483	1.213
Glomerular cell number (N)	1.000	0.378	0.999	1.000	1.000	0.681	0.999	1.001
Nonpodocyte glomerular cell number (N)	1.000	0.225	0.999	1.000	1.000	0.874	0.999	1.001
Podocyte number per glomerular tuft (N)	1.003	0.148	0.999	1.007	0.998	0.213	0.994	1.001
Glomerular cell density (N/10^6^ μm^3^)	0.999	0.648	0.993	1.005	1.010	**0.019**	1.002	1.018
Nonpodocyte glomerular cell density (N/10^6^ μm^3^)	0.996	0.213	0.989	1.003	1.012	**0.013**	1.003	1.021
Podocyte density (N/10^6^ μm^3^)	1.021	**0.040**	1.001	1.040	1.001	0.934	0.986	1.015
Nonsclerotic glomerular density (N/mm^2^)	2.403	**0.033**	1.072	5.386	0.768	0.469	0.376	1.569
Cortical fibrosis (μm^2^/μm^2^)	0.841	**0.006**	0.743	0.952	0.953	0.288	0.872	1.041

(-) Odds ratio or 95% CI could not be calculated because of a lack of events. Slow responders were defined as having a treatment response ≥16 weeks. Statistically significant *P* values (≤0.05) are stated in bold.

Abbreviations: CI, confidence interval; CR, complete remission; eGFR, estimated glomerular filtration rate; UPCR, Urinary protein creatinine ratio.

**Figure 3:**
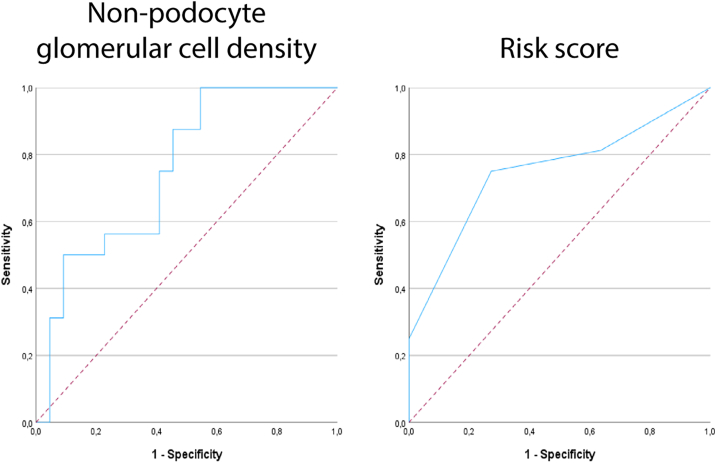
Receiver operating characteristic (ROC) curves for prognostic accuracy of time until remission in patients with idiopathic nephrotic syndrome (iNS). Nonpodocyte glomerular cell density was expressed as the mean nonpodocyte glomerular cell density. Areas under the curve were as follows: nonpodocyte glomerular cell density, 0.75 (95% CI, 0.598-0.907; *P* = 0.009); risk score for slow responders, 0.75 (95% CI, 0.582-0.918; *P* = 0.009). Dashed lines indicate the trendline. Abbreviations: CI, confidence interval

Next, we investigated associations between clinical or biopsy characteristics and time to remission. We stratified these patients based on the KDIGO guidelines^21^) for treatment response. Baseline clinical and experimental parameters were reported in Table 4. In total, 29 out of 47 patients showed treatment response ≤ 16 weeks (fast responders), and 18 out of 47 patients showed treatment response ≥ 16 weeks (slow/nonresponders). Interestingly, fast and slow responders were statistically significantly different in only 2 parameters, namely glomerular cell density and nonpodocyte glomerular cell density (Table 4). As expected, using logistic regression analysis, only glomerular cell density and nonpodocyte glomerular cell density were statistically significantly associated with delayed response (≥16 weeks) (Table 3). For nonpodocyte glomerular cell density, the OR was 1.012 (95% CI, 1.003-1.021; *P* = 0.01). At a threshold of N ≥ 425/10^6^ μm^3^, PPV and NPV were 83% and 77%, respectively. The ROC-AUC value was 0.75 (95% CI, 0.598-0.907; *P* = 0.009) (Fig 3). Conversely, when combining the percentage of nonsclerotic glomeruli, glomerular tuft volume, and nonsclerotic glomerular density into a risk score (Table 5), in part stratified based on our control data, we were able to predict a delayed response as well. Patients received points (maximum of 7) based on their individual morphometric values stratified into low, medium, and high risk (Table 5). For this risk score, the OR was 2.814 (95% CI, 1.267-6.253; *P* = 0.01). At a threshold for this risk score of ≥ 5 (out of 7), PPV and NPV were 67% and 80%, respectively. The ROC-AUC value was 0.75 (95% CI, 0.582-0.918; *P* = 0.009) (Fig 3).

**Table 4:** Baseline Clinical and Experimental Characteristics for Fast and Slow Responders in Patients With Idiopathic Nephrotic Syndrome (iNS)

Characteristic	iNS Fast Responders			iNS Slow Responders			*P* Value
	N	Median	IQR	N	Median	IQR	
Age (y)	29	63	45-72	18	52	34-66	0.123
Sex (% male)	29	55%		18	61%		0.689
Ethnicity (% White)	29	100%		18	100%		-
Serum creatinine concentration (μmol/L)	27	130	74-250	15	140	77-232	0.634
eGFR (mL/min/1.73 m^2^)	27	54	24-82	16	42	17-85	0.876
Serum albumin concentration (g/L)	27	20	16-29	14	18	11-25	0.154
UPCR (g/mmol)	24^a^	6.04	3.61-8.77	16^a^	7.38	4.62-10.82	0.570
Sclerotic glomeruli (% of total)	28	2.82	0.00-10.48	17	11.54	0.00-23.64	0.347
Nonsclerotic glomeruli (% of total)	28	97.18	89.52-100.00	17	88.46	73.37-100.00	0.347
Glomerular volume (10^6^ μm^3^)	22	5.7	4.5-7.2	16	5.5	4.0-6.0	0.265
Glomerular tuft volume (10^6^ μm^3^)	22	5.2	3.9-6.1	16	4.4	3.1-5.6	0.260
Glomerular cell number (N)	22	2630.4	2,120.3-3,122.3	16	3,017.5	1,741.0-3,433.5	0.690
Nonpodocyte glomerular cell number (N)	22	2082.1	1,814.4-2,678.3	16	2,631.4	1,471.5-2,906.2	0.878
Podocyte number per glomerular tuft (N)	22	380.1	230.4-596.2	16	340.9	274.0-431.7	0.181
Glomerular cell density (N/10^6^ μm^3^)	22	435.0	358.8-491.5	16	521.5	448.8-555.5	**0.008**
Nonpodocyte glomerular cell density (N/10^6^ μm^3^)	22	358.0	272.5-405.5	16	424.0	364.3-501.8	**0.005**
Podocyte density (N/10^6^ μm^3^)	22	82.50	55.0-129.0	16	84.0	56.8-122.8	0.936
Nonsclerotic glomerular density (N/mm^2^)	27	2.2	1.8-3.0	17	2.3	1.4-2.5	0.479
Cortical fibrosis (μm^2^/μm^2^)	28	0.172	0.132-0.223	18	0.154	0.083-0.200	0.289

Proteinuria was not always measured at the time of biopsy. For patients’ biopsies, all glomeruli were analyzed for biopsies with a glomerulus count of N ≥ 4. Continuous data are expressed as median (IQR). Student’s *t*-test or χ^2^-test was performed for statistical comparison. Statistically significant *P* values (≤0.05) are stated in bold.

Abbreviations: IQR, interquartile range; UPCR, urinary protein creatinine ratio.

a All patients had nephrotic range proteinuria at the time of presentation (≥ 3.5 g/10 mmol).

**Table 5:** Risk Score Categories for Slow Responders in Patients With Idiopathic Nephrotic Syndrome

Category	Low (1)	Medium (2)	High (3)
Nonsclerotic glomeruli (%)	≥ 90	≤ 89	
Glomerular tuft volume (10^6^ μm^3^)	≥ 5.1	5.0-3.3	≤ 3.2
Nonsclerotic glomerular density (N/mm^2^)	≥ 2.1	≤2.0	

The biopsy-based parameters, nonsclerotic glomeruli (%), glomerular tuft volume (10^6^ μm^3^), and nonsclerotic glomerular density (N/mm^2^) were combined into a risk score, stratified based on control data. Low, medium, and high risk elements were weighed as 1, 2, or 3 “scores,” respectively. Glomerular tuft volume was divided into 3 segments because of the extended range observed in biopsies of patients with primary nephrotic syndrome. Using a threshold of a combined 5 points (out of 7) or higher, the risk score could predict a delayed response. The odds ratio was 2.814 (95% CI, 1.267-6.253; *P* = 0.01).

#### Membranous nephropathy

In the MN cohort, patients had a median follow-up time of 50.1 months. During follow-up, the overall remission rate was 95% (41% PR and 54% CR). Median time to remission was 9.8 months. In total, 35 patients (59.3%) received IS treatment, the majority with combination therapy (47.5%) (Table 2). No statistically significant differences were observed in time to remission (*P* = 0.94) or treatment outcome (PR/CR%) between different treatment regimens (*P* = 0.83 and *P* = 0.84 for PR and CR, respectively). Patients with MN frequently reach remission with conservative treatment, without the use of IS medication.^7^^,^^31^ Patients who received IS treatment had lower serum albumin concentration than patients who received conservative treatment, indicating more severe nephrotic syndrome, whereas estimated glomerular filtration rate and the protein/creatinine ratio were not statistically different (Table 6). Additionally, IS-treated patients were more often PLA2R positive than conservatively treated patients (Table 6).

**Table 6:** Baseline Clinical and Experimental Characteristics of Immunosuppressive (IS) or Conservatively Treated Patients With Membranous Nephropathy (MN)

Characteristic	IS Treatment			No IS Treatment			*P* Value
	N	Median	IQR	N	Median	IQR	
Age (y)	35	57	42-63	24	60	49-70	0.134
Sex (% Male)	35	74%		24	88%		0.215
Ethnicity (% White)	36	100%		24	100%		-
Serum creatinine concentration (μmol/L)	34	93	81-116	20	91	79-105	0.506
eGFR (mL/min/1.73 m^2^)	35	77	58-91	22	81	63-93	0.397
Serum albumin concentration (g/L)	33	19	16.5-25.5	23	27	21.0-33.0	**0.001**
UPCR (g/mmol)	34^a^	6.23	4.07-7.95	22^a^	4.62	3.08-8.28	0.159
PLA2R positive (%)^b^	35	89%		24	58%		**0.007**
PLA2R serum titer (U/mL)^b^	21	91	48-179	8	55	14-120	0.113
Sclerotic glomeruli (% of total)	35	25.0	9.1-50.0	24	15.0	6.3-25.0	0.062
Nonsclerotic glomeruli (% of total)	35	75.0	50.0-90.9	24	85.0	75.0-93.8	0.062
Glomerular volume (10^6^ μm^3^)	32	6.3	4.5-8.3	23	6.7	4.9-7.3	0.405
Glomerular tuft volume (10^6^ μm^3^)^c^	13	7.0	4.6-8.2	19	5.0	4.1-6.6	0.071
Glomerular cell number (N)^c^	13	2,467.2	2,078.6-3,578.0	18	2218.6	1,614.7-2,746.1	0.058
Nonpodocyte glomerular cell number (N)^c^	13	2,108.1	1,926.1-3,143.1	18	2,007.3	1,413.0-2,364.4	0.057
Podocyte number per glomerular tuft (N)^c^	13	248.0	191.5-396.9	18	230.0	141.0-344.4	0.316
Glomerular cell density (N/10^6^ μm^3^)^c^	13	337.0	321.5-389.5	18	329.0	299.8-353.0	0.550
Nonpodocyte glomerular cell density (N/10^6^ μm^3^)^c^	13	286.0	272.5-334.0	18	282.0	262.8-304.3	0.557
Podocyte density (N/10^6^ μm^3^)^c^	13	48.0	37.5-53.0	18	43.5	30.5-62.8	0.861
Nonsclerotic glomerular density (N/mm^2^)	35	2.4	1.4-3.2	23	2.2	1.6-2.7	0.330
Cortical fibrosis (μm^2^/μm^2^)	29	0.170	0.122-0.214	23	0.173	0.130-0.246	0.733

Continuous data are expressed as median (IQR). Student’s *t*-test or χ^2^-test was performed for statistical comparison. Statistically significant *P* values (≤0.05) are stated in bold.

Abbreviations: eGFR, estimated glomerular filtration rate; IQR, interquartile range; PNS, primary nephrotic syndrome; UPCR, urinary protein creatinine ratio.

a All patients had nephrotic range proteinuria at the time of presentation (≥ 3.5 g/10 mmol). Proteinuria was not always measured at the time of biopsy.

b In older cases, the PLA2R serum titer was not available for MN.

c In older biopsies, the use of Bouin’s fixative prevented immunofluorescent stainings, and subsequent parameters could not be computed. Contemporary biopsies fixed with formalin could be analyzed. For patients’ biopsies, all glomeruli were analyzed for biopsies with a glomerulus count of N ≥ 4. *t* test was performed for statistical comparison.

**Figure 4:**
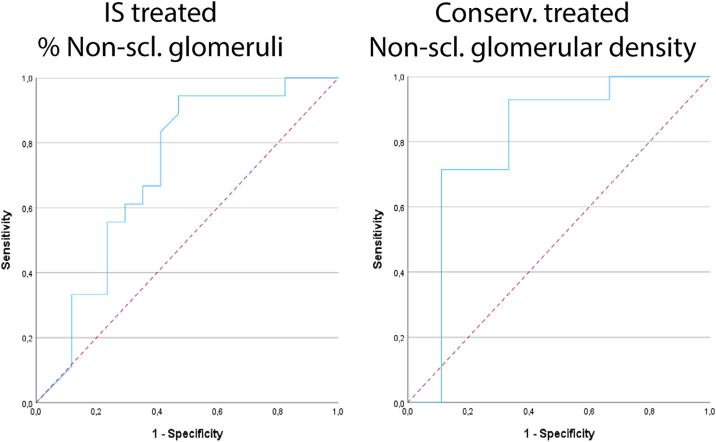
Receiver operating characteristic (ROC) curves for prognostic accuracy of reaching complete remission (CR) in patients with membranous nephropathy (MN). Nonsclerotic glomerular density was expressed as mean nonsclerotic glomerular density. Areas under the curve were as follows: Immunosuppressive-treated MN percentage OF nonsclerotic glomeruli for prediction of complete remission (CR), 0.71 (95% CI, 0.535-0.893; *P* = 0.03); Conservatively treated nonsclerotic glomerular density for prediction of CR, 0.80 (95% CI, 0.584-1.000; *P* = 0.01). Dashed lines indicate the trendline. Abbreviations: CI, confidence interval; IS, immunosuppressive; Conserv., conservatively

We investigated whether any of the clinical or biopsy-based parameters could predict CR in patients who received either conservative or IS treatment. No statistically significant differences in follow-up duration were observed between patients who reached CR versus those who did not reach CR in patients who received IS treatment and conservative treatment (*P* = 0.36 and *P* = 0.38, respectively). Using logistic regression analysis, the percentage of nonsclerotic glomeruli and nonsclerotic glomerular density were significantly associated with the outcome of CR, both in patients who received IS treatment and patients who received conservative treatment (Table 7). For the percentage of nonsclerotic glomeruli, the OR for CR was 1.040 (95% CI, 1.006-1.075; *P* = 0.02). At a threshold for the percentage of nonsclerotic glomeruli of ≥ 75%, PPV and NPV values were 67% and 65%, respectively. For nonsclerotic glomerular density, the OR for CR was 6.732 (95% CI, 1.271-35.648; *P* = 0.02). At a threshold for nonsclerotic glomerular density of ≥ 1.9 N/mm^2^, PPV and NPV values were 79% and 67%, respectively. ROC-AUC values were 0.71 (95% CI, 0.535-0.893; *P* = 0.03) and 0.80 (95% CI, 0.584-1.000; *P* = 0.01) for the percentage of nonsclerotic glomeruli and nonsclerotic glomerular density, respectively (Fig 4).

**Table 7:** Logistic Regression Analysis for Predicting CR in Patients With Membranous Nephropathy (MN)

Characteristic	CR IS 95% CI				CR Cons. 95% CI			
	Odds Ratio	*P* Value	Lower	Upper	Odds ratio	*P* Value	Lower	Upper
Age (y)	1.043	0.124	0.988	1.100	1.032	0.364	0.964	1.106
Sex (% Male)	0.210	0.081	0.036	1.210	-	-	-	-
Ethnicity (% White)	-	-	-	-	-	-	-	-
Serum creatinine concentration (μmol/L)	0.996	0.656	0.979	1.013	0.992	0.510	0.969	1.016
eGFR (mL/min/1.73 m^2^)	0.989	0.419	0.963	1.016	0.988	0.558	0.948	1.029
Serum albumin concentration (g/L)	1.046	0.468	0.926	1.182	1.102	0.162	0.962	1.262
UPCR (g/mmol)	0.969	0.708	0.820	1.144	0.918	0.611	0.659	1.278
PLA2R positive (Yes/No)	0.313	0.336	0.029	3.344	0.429	0.332	0.077	2.371
PLA2R serum titer (U/mL)	0.991	0.081	0.981	1.001	1.064	0.149	0.978	1.158
Sclerotic glomeruli (% of total)	0.962	**0.022**	0.930	0.994	0.955	0.125	0.901	1.013
Nonsclerotic glomeruli (% of total)	1.040	**0.022**	1.006	1.075	1.047	0.125	0.987	1.109
Glomerular volume (10^6^ μm^3^)	1.099	0.501	0.835	1.445	1.172	0.561	0.687	2.001
Glomerular tuft volume (10^6^ μm^3^)	1.311	0.285	0.798	2.154	0.877	0.683	0.466	1.649
Glomerular cell number (N)	1.000	0.897	0.999	1.001	1.000	0.905	0.998	1.002
Nonpodocyte glomerular cell number (N)	1.000	0.988	0.999	1.001	1.000	0.839	0.998	1.002
Podocyte number per glomerular tuft (N)	1.007	0.191	0.996	1.018	1.002	0.726	0.993	1.010
Glomerular cell density (N/10^6^ μm^3^)	0.998	0.867	0.979	1.018	0.995	0.499	0.980	1.010
Nonpodocyte glomerular cell density (N/10^6^ μm^3^)	0.997	0.820	0.975	1.020	0.988	0.334	0.963	1.013
Podocyte density (N/10^6^ μm^3^)	1.006	0.883	0.924	1.097	1.021	0.379	0.975	1.070
Nonsclerotic glomerular density (N/mm^2^)	1.319	0.295	0.786	2.212	6.732	**0.025**	1.271	35.648
Cortical fibrosis (μm^2^/μm^2^)	1.036	0.521	0.930	1.154	0.869	0.072	0.745	1.013

(-) The odds ratio or 95% confidence interval could not be calculated because of a lack of events. Slow responders were defined as having a treatment response ≥ 26 weeks. Statistically significant *P* values (≤0.05) are stated in bold.

CI, confidence interval; CR, complete remission; cons, conservatively treated (without the use of immunosuppressive drugs); eGFR, estimated glomerular filtration rate; IS, immunosuppressive; UPCR, urinary protein creatinine ratio.

## Discussion

Morphometrics, the study of spatial distribution and numbers (ie, nephron number or podocyte number), has been shown to be valuable for both diagnostic and prognostic purposes. Many studies have shown associations between biopsy-based parameters and disease chronicity, disease outcome, or treatment response in various glomerular diseases.^32^ Complementary to this, others have investigated the associations between clinical parameters and treatment outcome.^33^, ^34^, ^35^, ^36^, ^37^ Despite the potential clinical relevance of the podocyte depletion hypothesis as a conceptual framework, few have reported on the association of podocyte indices or glomerulosclerotic indices and long-term renal outcomes in PNS, especially on the predictive value at the individual level. By the quantitation of biopsy morphometrics, this study is the first to systematically compare cortical, glomerular, and podocyte indices in patients with PNS and shows several biopsy-derived parameters that predicted CR on an individual level in patients with PNS. For iNS, we identified the percentage of nonsclerotic glomeruli (≥95%), podocyte density (N/10^6^ μm^3^ ≥ 80), nonsclerotic glomerular density (N/mm^2^ ≥ 2.2), and cortical fibrosis (≤19.5%) as predictors. Furthermore, nonsclerotic glomerular cell density and a biopsy-based parameter risk score of multiple biopsy-based parameters (percentage of nonsclerotic glomeruli, glomerular tuft volume, and nonsclerotic glomerular density) both predicted delayed remission (≥16 weeks). In patients with MN, the percentage of nonsclerotic glomeruli (≥75%) and nonsclerotic glomerular density (N/mm^2^ ≥ 1.9) predicted CR.

According to the podocyte depletion hypothesis, the outcome of any glomerular injury depends on the depletion of the remaining podocyte pool. In response, in vivo human podocytes become hypertrophic as a compensation mechanism for sufficient podocyte loss.^38^ Interestingly, many patients in our study show podocyte number/density levels far below control levels. Healthy adult glomeruli contain around 600 podocytes, although this number depends on age.^4^^,^^39^, ^40^, ^41^ In the rat model of Wharram et al,^8^ it is suggested that sclerotic lesion formation may already initiate from ≥20% podocyte loss. For humans, given enough time for compensatory mechanisms, more podocyte depletion may be endured without causing any glomerulosclerosis.^39^ Recently, de Zoysa et al^42^ performed similar studies in a cohort of patients with FSGS. It was shown that podocyte number (N ≥ 216) and the percentage of sclerotic glomeruli (≥50%) at biopsy differentiated between treatment responders and nonresponders at 6 months after disease onset. Although we found these parameters to be predictive of CR and not time until remission, these data together demonstrate that quantitation of podocyte depletion and glomerulosclerosis in PNS biopsies has prognostic value. Notably, our iNS cohort also included patients with MCD, suggesting that the predictive value of podocyte number and the amount of (global) glomerulosclerosis applies not only to patients with FSGS.

Previously, Tsuboi et al^25^ reported that glomerular density (including focal but excluding globally sclerotic glomeruli) was significantly associated with disease progression (≥ 50% reduction of estimated glomerular filtration rate) in patients with MN. Coupled with the data of Tsuboi et al,^25^ our findings show the prognostic potential of morphometric analyses in patients with MN. Podocyte depletion, although widely present in most of our patients with MN, could not predict CR. Interestingly, podocyte density was, on average, much lower compared with iNS, even though patients with MN could still reach CR. We speculate that patients with reduced podocyte count may still have had sufficient functioning glomeruli to achieve potential CR, because healthy adult controls’ kidneys have been described with very low nephron numbers without any renal complications.^43^ We were unable to estimate nephron number in our patient cohorts because computed tomography or magnetic resonance imaging scanning (to assess cortical volume) was not routinely performed. We used glomerular density as an estimate of total nephron number.^44^ In patients who had more severe podocyte depletion and glomerulosclerosis, nonsclerotic glomerular density, or the percentage of nonsclerotic glomeruli, may therefore better represent the remaining pool of functioning glomeruli compared with podocyte number/density.

It is well established that podocyte and glomerular-derived parameters vary between healthy individuals, depending on age, gender, race, weight, and zonal location within the kidney.^14^^,^^24^^,^^29^^,^^39^^,^^45^ Adjusting biopsy-derived parameters for these confounders could further personalize their use for routine care. Owing to the relatively small cohorts included in this study, we were unable to reliably include them as covariates during predictive modelling. Additionally, some patients lacked data for some experimental parameters, such as podocyte number, (nonsclerotic) glomerular density, or glomerular (tuft) volume, because they did not meet biopsy inclusion standards for minimal glomerular count for all biopsy sections (N ≥ 4). Clinically, patients with glomerular counts ≤4 were similar to patients with glomerular counts ≥ 4. However, we cannot exclude the possibility that, if included, the patients most at risk (through intrinsic low glomerular densities and therefore low biopsy glomerular count) would yield slightly different results. Furthermore, we were unable to measure biopsy-based parameters (ie, podocyte number/density) in some older biopsies (only relevant in our MN cohort) because the used tissue fixative interfered with our immunofluorescent staining. The inherent loss of statistical power for these parameters could mean that we potentially did not detect their predictive value.

IS treatment is considered first-line therapy in patients with iNS. For patients with MN, the initiation of IS treatment is guided by timed urinary measurements used to predict disease progression,^20^ because spontaneous remission occurs frequently.^7^^,^^31^ No statistically significant differences were observed between treatment outcome and (IS or conservative) treatment regimen.

In conclusion, our findings supported our hypothesis that podocyte depletion determines treatment outcome in patients with iNS. Higher podocyte density and nonsclerotic glomerular density, and less glomerulosclerosis and cortical fibrosis at biopsy could predict CR in patients with iNS. In patients with MN who received IS treatment, less glomerulosclerosis predicted CR. Additionally, higher nonsclerotic glomerular density predicted CR in patients with MN who were treated conservatively. Morphometric analysis of kidney biopsies may be of prognostic clinical value in patients with PNS. Admittedly, CIs in our study were wide, which prevents an accurate assessment of the prognostic value of our findings. Therefore, we propose that morphometric analysis of biopsy specimens should be implemented in registries and intervention studies to validate these findings.

## Supplementary Material

Supplementary File (PDF) Figures S1-S2; Item S1; Tables S1-S2
