# Supplementary material for: Biopsy Morphometrics as Predictors of Treatment Response in Primary Nephrotic Syndrome
Source: Kidney Med. 2025 Nov 7;8(1):101181. doi: 10.1016/j.xkme.2025.101181 (PMC12774748; doi:10.1016/j.xkme.2025.101181)
Supplement: Supplementary File (PDF) — Figures S1-S2; Item S1; Tables S1-S2 [file mmc1.pdf]

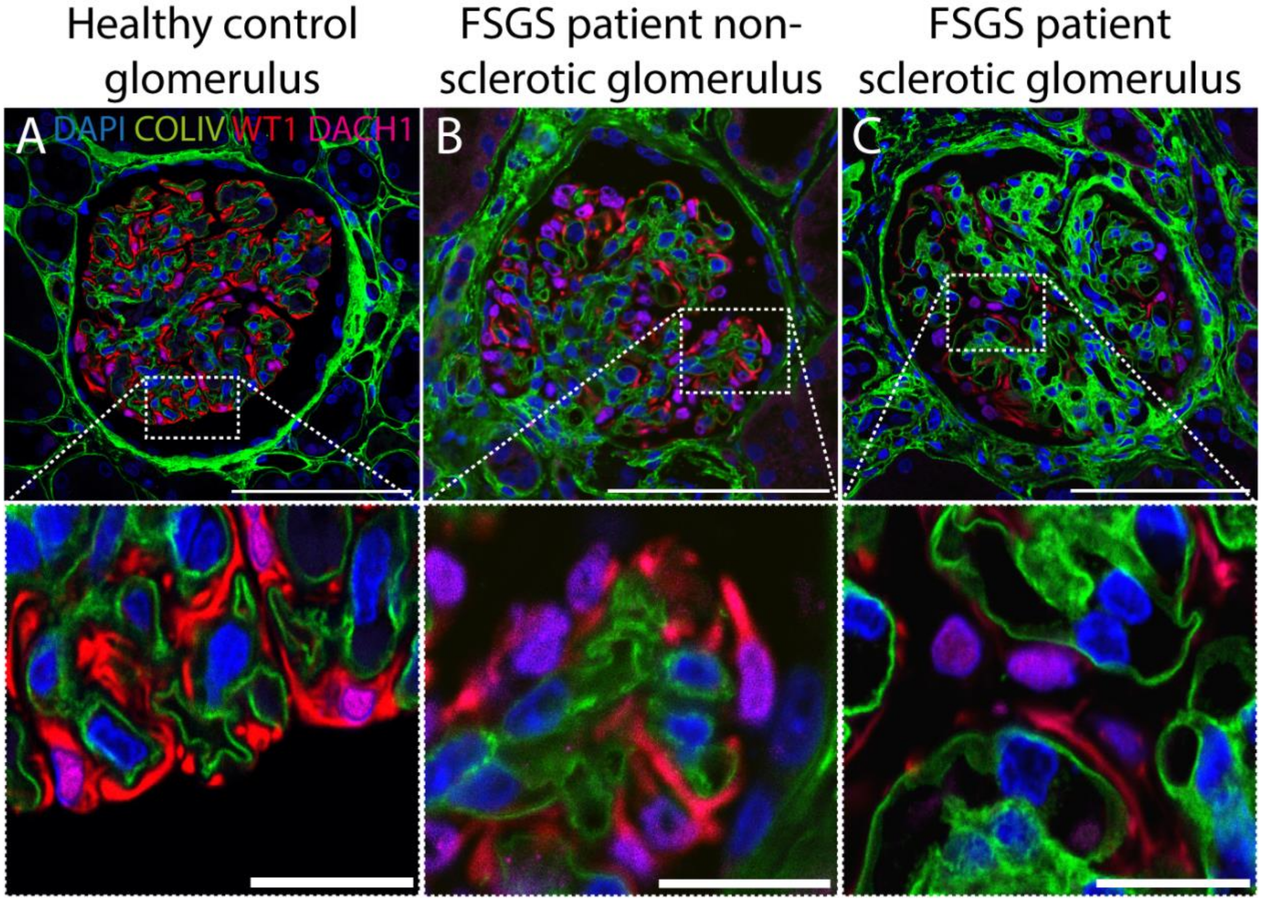

Figure S1. Overview of podocyte-specific cytoplasmic and nuclear antigens. Representative images of a healthy glomerulus, unaffected glomerulus from a FSGS patient and affected (FSGS lesion) glomerulus from the same FSGS patient. Biopsy tissue was stained for DAPI (nuclei, blue), collagen type IV (COLIV, fibrous tissue and basement membranes, green), Wilms' tumor 1 (WT1, podocyte-specific cytoplasm, red) and Dachshund family transcription factor 1 (DACH1, podocyte nuclei, magenta). All DAPI$^+$/DACH1$^+$ nuclei were surrounded by cytoplasmic WT1. In severely affected glomeruli, WT1 and DACH1 expression levels did decrease, indicative of a gradual process before actual podocyte loss. Scale bars represent 100 µm (upper part) and 20 µm (lower part), respectively.

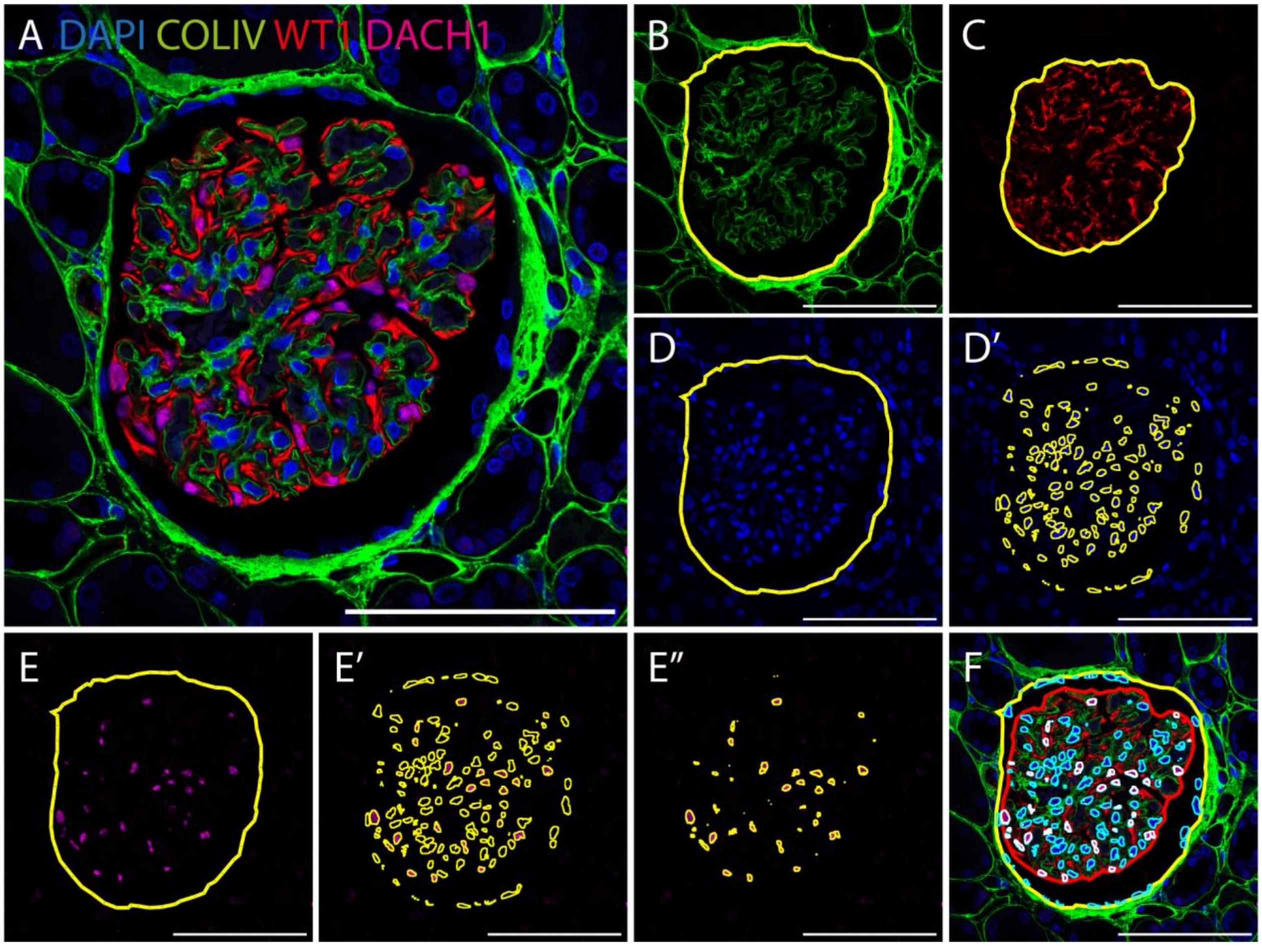

Figure S2. Overview of podocyte and glomerular annotations. (A) Representative overview of a glomerulus from a healthy control stained for DAPI (nuclei, blue), collagen type IV (COLIV, fibrous tissue and basement membranes, green), Wilms’ tumor 1 (WT1, podocyte-specific cytoplasm, red) and Dachshund family transcription factor 1 (DACH1, podocyte nuclei, magenta). (B) Glomerular annotation of Bowman’s capsule (yellow) using COLIV (green) as reference image. (C) Glomerular tuft annotation (yellow) using podocyte-specific cytoplasmic WT1 (red) as reference image. (D-D’) Glomerular nuclei annotation using the COLIV glomerular annotation (B) as a selection (D) and then thresholded DAPI (D’). (E-E”) Podocyte nuclei annotation using the COLIV glomerular annotation (B) and glomerular DAPI annotation (D’) as selections (E and E’, respectively) and then thresholded DAPI^{+} DACH1 (E”). (F) Overview of all glomerular (Bowman’s capsule, yellow), glomerular tuft (red), glomerular nuclei (cyan) and podocyte nuclei (white) annotations. Scale bars represent 100 µm.

Item S1. Supplementary Materials and Methods

Clinical outcome definitions

Patients were considered in complete remission (CR) if proteinuria was ≤0.3 g/10 mmol. Patients were considered in partial remission if proteinuria was between ≥0.3 g/10 mmol and ≤3.5 g/10 mmol or a decrease was observed of ≥50% from onset PNS and proteinuria was ≤3.5 g/10 mmol. Relapse was defined as proteinuria of ≥3.5 g/10 mmol after partial or complete remission had been achieved. Distinction between fast and slow responders to treatment was based on the KDIGO guidelines and set to 16 weeks after disease onset for both MCD and FSGS.

Spontaneous remission is described as obtaining (partial or complete) remission without the use of immunosuppressive treatment. Routine treatment for MN has varied significantly over time. Since spontaneous remission occurs frequently in MN (Hofstra 2011. CJASN; Polanco 2010. JASN), our university medical center routinely employs timed urinary measurements to predict outcome of symptomatic treatment (van den Brand 2011. CJASN), which guide clinical decision making. Knowledge regarding optimal immunosuppressive treatment regimen was updated several times during the treatment of our included patients, leading to the heterogeneous immunosuppressive treatment regimen administered.

Conservative treatment otherwise known as supportive care in glomerular disease is the management of edema, hypercoagulability and thrombosis, hyperlipidemia, hypertension and proteinuria reduction when needed (KDIGO 2021 Clinical practice guideline for the management of glomerular disease. 2021 Kidney International).

All relevant PNS patients' clinical data was collected by accessing electronic medical records, which included the pathology report for biopsy-confirmed diagnosis. Collected relevant information included (but is not limited to) age at time of biopsy, sex, ethnicity, serum or urinary creatinine at time of biopsy

and follow up time points when available, pathologist's report and immunosuppressive treatment regimen received.

Immunofluorescent staining

Paraffin embedded kidney biopsy material of patients with PNS and controls was deparaffinized using a series of xylol (2x) and 100% (v/v) ethanol (3x). Tissue slides were rinsed in demineralized water once after which antigen retrieval was performed by boiling tissue slides in their respective buffers for 3 min at 600 W and another 60 min at 180 W. Tissue slides were cooled down on ice for 20 min and blocked with 10% donkey blocking serum in PBS (DBS, Gibco) for 10 min. Primary (1:100) and secondary (1:200) antibodies were diluted in PBS containing 1% (v/v) bovine serum albumin (BSA, Sigma). Primary antibodies were incubated overnight at 4°C and secondary antibodies at room temperature for 2 hours. Between and after antibody incubations, tissue slides were washed for 10 min in their respective buffers. Tissue slides were mounted with Fluormount-G® (Southern Biotech, SanBio) and covered with glass cover slips. Biopsy tissue was stained for DAPI (nuclei), Collagen type IV (COLIV, fibrous tissue and basement membranes), Wilms' tumor 1 (WT1, podocyte-specific cytoplasm) and Dachshund family transcription factor 1 (DACH1, podocyte nuclei) Dach1 was previously shown to be highly expressed and specific to podocytes in the glomerulus (Endlich *et al.* J Cell Mol Med 2018. The transcription factor Dach1 is essential for podocyte function) and used by others for similar purposes (de Zoysa *et al.* Front Med (Lausanne). Podocyte number and glomerulosclerosis indices are associated with the response to therapy for primary focal segmental glomerulosclerosis, Haruhara *et al.* J Am Soc Nephrol 2021. Podometrics in Japanese Living Donor Kidneys: Associations with Nephron Number, Age, and Hypertension). Images were captured using a Zeiss LSM 880 confocal microscope or the 3DHistech Panoramic Midi II immunofluorescence slide scanner. In older biopsies in our university medical center (< 2016), the use of bouin's fixative prevented successful immunofluorescent staining of DACH1 and WT1.

Calculation of parameters dependent on these immunofluorescent stainings could not be performed in some patients. Contemporary biopsies (> 2016), fixed with formalin could be used successfully.

Podometric, glomerular and cortical analyses

Several custom-made and semi-automated macro scripts (Fiji) were created for the consistent and (relative) high throughput quantification of podocyte and glomerular specific properties and general cortical fibrosis in kidney biopsies (10.5281/zenodo.13122255). For patients' biopsies, all glomeruli were analyzed for biopsies with a glomerulus count of N ≥ 4 as this was indicated by Puelles *et al.* (Nephrol Dial Transplant. 2022. Estimating individual glomerular volume in the human kidney: clinical perspectives), to result in little variation within and between cohorts when compared to larger glomeruli counts (up to 30). For control tissue obtained from the healthy part of nephrectomy material, at least 10 cortical glomeruli were analyzed for each of the following parameters. Some parameters could not be calculated in all patients, because they lacked a sufficient glomerulus count (N ≥ 4). All patients included had at least one biopsy-based parameter that could be calculated.

<u>Podocyte density and number</u>

Podocyte density was calculated based on the method described by Venkatareddy *et al.* (2014. JASN). Fluorescent images obtained of diamidinophenylindole (DAPI), collage type IV (COLIV), Wilms' tumor 1 (WT-1, cytoplasmic) and Dachshund Family Transcription Factor 1 (DACH1) were used for this end. All $DAPI^+/DACH1^+$ nuclei were surrounded by cytoplasmic WT-1^+, indicating correct podocyte detection for quantification (Supplementary figure S1). Glomerular and glomerular tuft area were manually annotated using COLIV and WT-1 for glomerular and podocyte specific (tuft) area, respectively. Using COLIV as a selection for the glomerulus, $DAPI^+/DACH1^+$ podocyte nuclei were counted and mean caliper diameter was measured. Podocyte density was calculated by using podocyte count (N ($DAPI^+/DACH1^+$)), mean apparent nuclear caliper diameter ($DAPI^+/DACH1^+$ nuclei), glomerular tuft area (WT-1 circumference,

um^2) and optical section thickness. An overview of podocyte and glomerular annotations can be found in supplementary figure 2. For optical section thickness, we used the formula for axial resolution on confocal microscopes.

$$\text{Axial resolution} = (1.4 \times \lambda \times \eta)/(NA^2)$$

λ is used to describe the emitted light wavelength in nm (647 nm). η represents the refraction index (1.338 for air). NA (Numerical Aperture) is a dimensionless number that describes the range of angles over which the used objective and microscope can detect/emit light (0.8 for the objective used). Since we imaged all glomeruli with the same objective and used the same staining protocol (meaning DACH1 was always stained with a secondary 647 antibody), our axial resolution became a constant of 1.9 µm (rounded to a single decimal).

Podocyte number per glomerular tuft was calculated by multiplying podocyte density (N x 10^6 μm^3) with glomerular tuft volume (10^6 μm^3) (see below).

<u>Glomerular cell density and number</u>

Glomerular cell density was calculated similar to the method described above for podocyte density quantification. Fluorescent images obtained of DAPI, COLIV, WT-1 (cytoplasmic) and DACH1 were used for this end. Instead of counting DAPI$^+$/DACH1$^+$ podocyte nuclei, glomerular DAPI$^+$ nuclei were counted. Glomerular cell density was calculated using glomerular volume (COLIV) instead of glomerular tuft volume (WT-1)(to include parietal epithelial cells in this parameter). Non-podocyte glomerular cell density was calculated by subtracting podocyte density from glomerular cell density. (Non-podocyte) glomerular cell number per glomerulus was calculated by multiplying (non-podocyte) glomerular cell density (N x 10^6 μm^3) with glomerular volume (10^6 μm^3) (see below).

<u>Sclerotized glomerular percentage</u>

The percentage of (non-)sclerotic glomeruli was calculated by counting the amount of (non-)sclerotic glomeruli divided by the total amount of glomeruli. The percentage of sclerotic glomeruli includes both segmentally and globally sclerotized glomeruli.

Glomerular (tuft) volume

Biopsies were analyzed for glomerular volume and glomerular tuft volume, identical to the glomerular tuft annotations we used in the method of Venkatareddy *et al.* for our podocyte density calculations. Glomerular (tuft) area was assessed on fluorescent images obtained of DAPI, COLIV, WT-1 (cytoplasmic) and DACH1 and was defined as either the area on the inner side of Bowman's capsule (glomerular volume, based on COLIV) or on the outer side of the capillary loops of the glomerular tuft (glomerular tuft volume, based on WT-1 (circumference)). For sclerotic glomeruli, sclerotic lesions were not included in glomerular tuft area since those lacked both cytoplasmic WT-1 expression as well as $DAPI^+/DACH1^+$ podocyte nuclei. Mean glomerular (tuft) area was used to calculate mean glomerular (tuft) volume using the Weibel and Gomez equation (1966. J Cell Biol).

$$\text{Glomerular (tuft) volume} = \beta/d \times (\text{mean glomerular (tuft) area})^{3/2}$$

B is a dimensionless shape coefficient (1.382 for spheres) and d is a size distribution coefficient (1.01) used to adjust for variations in glomerular size, previously used by Denic *et al.* (2017. JASN).

Glomerular density

Total cortical area per biopsy and total glomerular area per biopsy were annotated based on fluorescent images obtained of DAPI, COLIV, WT-1 (cytoplasmic) and DACH1 or a Periodic Acid-Schiff stain (PAS, in the case of older Bouin's fixated MN biopsies) using CaseViewer (3DHistech Ltd, version 2.4). All (non-sclerotic) glomeruli were counted. (Non-sclerotic) glomerular density was calculated by dividing the total number of (non-sclerotic) glomeruli (N) by the total cortical area (mm^2).

$$N \text{ ((non-sclerotic) glomeruli) / cortical area } (mm^2)$$

CAB matrix deposition as a marker for fibrosis

Similar to the estimated amount of (interstitial) fibrosis determined by the pathologist, we strived to fully quantify the amount of fibrosis present. Chromotrope Aniline Blue (CAB) stains all collagenous (matrix) tissue blue (as well as all basement membranes) and may therefore be used as a marker for the amount of fibrosis present in a kidney biopsy. Muscle and cellular cytoplasm stains red. Total cortical area per biopsy was manually annotated using CAB staining. Secondly, red signal was deducted from all images in order to get blue specific signal. Blue signal area was selected and the ratio of blue signal area per total cortical area was calculated.

$$\text{Blue signal area } (\mu m^2) \text{ / cortical area } (\mu m^2)$$

Table S1. Reagent and resource table

**Deposited Data**		
Scripts and codes for data analysis	This paper; deposited on Zenodo	10.5281/zenodo.13122255
**Software and Algorithms**		
ImageJ version Fiji 1.51n	National Institutes of Health, USA	https://imagej.nih.gov/ij
Adobe Illustrator CC 2021	Adobe Systems Inc.	RRID:SCR_010279
Adobe Photoshop CC 2021	Adobe Systems Inc.	RRID:SCR_014199
SPSS version 29	IBM SPSS statistics 29	RRID:SCR_016479
CaseViewer version 2.4	3DHistech	RRID:SCR_017654

Table S2. Immunofluorescent staining overview of used chemicals, antibodies, working dilutions and antigen retrieval buffers.

**Primary antibody or chemical**	**Working dilution**	**Secondary antibody**	**Working dilution**	**Antigen retrieval buffer**
Podocyte and glomerular morphometrics staining				
DAPI (D1306, Invitrogen)			1:1000	TBE
Goat Anti-Type IV Collagen-UNLB (1340-01, Southern Biotech)	1:100	Donkey anti-Goat Alexa Fluor™ 488 (A-11055, Thermo Fisher)	1:200	
Recombinant Anti-Wilms Tumor Protein antibody (ab89901, Abcam)	1:100	Donkey anti-mouse Alexa Fluor™ 568 (A10037, Thermo Fisher)	1:200	
Dachshund 1 (HPA012672, Sigma-Aldrich)	1:100	Donkey anti-rabbit Alexa Fluor™ 647 (A-31573, Thermo Fisher)	1:200	
